# Phospholipase Cγ2 is critical for Ca^2+^ flux and cytokine production in anti-fungal innate immunity of human corneal epithelial cells

**DOI:** 10.1186/s12886-018-0847-6

**Published:** 2018-07-13

**Authors:** Xudong Peng, Guiqiu Zhao, Jing Lin, Jianqiu Qu, Yingxue Zhang, Cui Li

**Affiliations:** 1 Department of Ophthalmology, The Affiliated Hospital of Qingdao University, NO. 16 Jiangsu Road, Qingdao, 266003 Shandong Province China; 2 Department of Biochemistry, Immunology and Microbiology, Wayne State University School of Medicine, 540 E. Canfield Avenue, Detroit, MI 48201 USA

**Keywords:** PLCγ, Dectin-1, Ca^2 +^, Innate immunity, Corneal epithelium, *Aspergillus fumigatus*

## Abstract

**Background:**

Fungal keratitis (FK) is a sight-threatening disease, accounting for a significant portion with its complex presentation, suboptimal efficacy of the existing therapies and uncontrollable excessive innate inflammation. Phospholipase C-γ2 (PLCγ2) is a non-receptor tyrosine kinase that plays an important role at the early period of innate immunity. This study aimed to identify the role of PLCγ2 in Dectin-1-mediated Ca^2+^ Flux and its effect on the expression of proinflammatory mediators at the exposure to *Aspergillus fumigatus* (*A. fumigatus*) hyphae antigens in human corneal epithelial cells (HCECs).

**Methods:**

The HCECs were preincubated with or without different inhibitors respectively before *A. fumigatus* hyphae stimulation. Intracellular calcium flux in HCECs and levels of PLCγ2 and spleen-tyrosine kinase (Syk) were detected by fluorescence imaging and Western Blotting. The expression of proinflammatory mediators was determined by reverse transcriptase polymerase chain reaction (RT-PCR) and enzyme-linked immunosorbent assay (ELISA).

**Results:**

We demonstrated that an intracellular Ca^2+^ flux in HCECs was triggered by *A. fumigatus* hyphae and could be reduced by pre-treatment with PLCγ2-inhibitor U73122. *A. fumigatus* hyphae induced PLCγ2 phosphorylation was regulated by Dectin-1 via Syk. Furthermore, PLCγ2-deficient HCECs showed a drastic impairment in the Ca^2+^ signaling and the secretion of IL-6, CXCL1 and TNF-α.

**Conclusions:**

PLCγ2 plays a critical role for Ca^2+^ Flux in HCECs stimulated by *A. fumigatus* hyphae. Syk acts upstream of PLCγ2 in the Dectin-1 signaling pathway. The expressions of proinflammatory mediators induced by *A. fumigatus* are regulated by the activation of Dectin-1-mediated PLCγ2 signaling pathway in HCECs.

## Background

FK is a sight threatening disorder associated with multiple risk factors, such as ocular surface disease, extended wear contact lenses, and traumatic ocular surface accidents [1], presenting a therapeutic challenge due to the lack of effective antifungal agents and uncontrollable excessive innate inflammation. Innate immunity is an important defence against microbial infections in cornea. However, excessive innate inflammatory response could damage normal corneal epithelial cells whilst slowing down the pathological progress of FK [2].

On one hand, cytoplasmic Ca^2+^ flux has been demonstrated to play an important role in innate immune response. In airway epithelia, cytoplasmic Ca^2+^ regulates *P. aeruginosa-* or *flagellin-* activated innate immune responses [3]. Additionally, cytoplasmic Ca^2+^ flux promotes macrophage to recognize carbohydrate structures on pathogenic fungi via C-type lectin receptors (CLRs) [4–7]. In lymphocytes, cytoplasmic Ca^2+^ flux is one of the hallmarks of B cell receptor (BCR) signaling [8, 9], in which the enzymatic activity of PLCγ2 is enssential for the induction of Ca^2+^ flux [10]. PLCγ2 is a non-receptor tyrosine kinase, playing an important role at the early period of innate immunity. It is documented that PLCγ2 is the key component in Dectin-2 signaling pathway, mediating anti-fungal innate immune responses in macrophages [11]. However, there is little evidence showing the relationship between PLCγ2 and the induction of Ca^2+^ flux in human corneal epithelial cells (HCECs) [12].

On the other hand, pattern-recognition receptors (PRRs) exert the regulatory role in innate immune response [2, 13]. At the early period of fungal infection, the innate immune system recognizes pathogen associated molecular pattern (PAMP) of pathogenic microorganism using PRRs, such as C-type lectin receptors (CLRs), Toll-like receptors (TLRs) and NOD-like receptors (NLRs) [14–17]. Dectin-1 is a CLR that can identify β-glucan of fungal cytoderm, mediating a variety of fungal innate immune responses and triggers signal transduction via its cytoplasmic hemi-ITAM [18]. The phosphorylated ITAM-like motif of Dectin-1 could directly recruit Syk, which subsequently signals downstream to activate mitogen-activated protein kinases (MAPKs) and nuclear factor κB (NF-κB). Additionally, Syk plays a significant role in Dectin-1 mediated signaling pathway as an antigen-receptor-like manner in magrophages [19, 20]. However, the function of Syk in the Dectin-1 signal pathway in HCECs is still unclear. Recently, we reported that Dectin-1 induced cytoplasmic Ca^2+^ flux upon *A. fumigatus* infection in HCECs [21], suggesting a potential relationship between PLCγ2 and Dectin-1, as well as cytoplasmic Ca^2+^ flux.

In this study, we demonstrated that the participation of Dectin-1 regulated the expression of PLCγ2 via Syk upon the treatment of *A. fumigatus* in HCECs. Moreover, PLCγ2 is the critical phospholipases in the process of Dectin-1-mediated Ca^2+^ flux and the secretion of proinflammatory mediators (IL-6, CXCL1 and TNF-α) in HCECs.

## Methods

### Materials and reagents

RNAiso Plus and RT-PCR kits were from TaKaRa (Dalian, China). RIPA (radioimmunopreci- pitation assay) was from Solarbio (Beijing, China). The BCA Protein Assay Kit, polyvinylidene difluoride (PVDF) membranes, confining liquid and enhanced chemiluminescence (ECL) kit were from Beyotime Biotechnology (Shanghai, China). The following reagents were purchased: PLCγ2 inhibitor-U73122 (MilliporeSigma, MO, USA), syk inhibitor-Piceatannol(Selleck, Texas, USA)and Dectin-1 inhibitor-Laminarin (MilliporeSigma, MO, USA). Antibodies used for confocal microscope were from AAT Bioquest (California, USA). Antibodies used for Western blot were from Cell Signaling(Danvers, MA): anti-PLCγ2, anti-phospho-PLCγ2 (Tyr759), anti-Syk, anti-phospho-Syk.

### The preparation of *A. fumigatus* suspension

*A.fumigatus* standard strain (CPCC 3.0772) was cultured in Sabouroud liquid culture at 37 °C 200 rpm for 2–3 days, and then the harvested mycelia of Aspergillus fumigatus was washed twice by sterile phosphate buffere saline (PBS) and sterilized by 70% ethanol at 4 °C for 12 h. The density of the fungal mycelia was read in a blood cell counting board and reached the final concentrations of 1 × 10^8^ colony-forming units per 1 ml. The inactived *A. fumigatus* mycelia was stored at − 20 °C [22, 23].

### Cell culture

HCECs were kindly offered by Ocular Surface Laboratory of Xia Men Eye Center and grown in DMEM/F12 with 6.4% Fetal bovine serum (FBS), 7.52 ng/ml Insulin, 7.52 μg/ml Epidermal Growth Factor (EGF), 100u/ml penicillin G and 100μg/ml streptomycin sulfate in a humidified 5% CO2 incubator at 37 °C. The medium was replaced every 2 days before experiments. HCECs suspensions of 1 × 10^5^/ml were seeded onto 12- or 6-well tissue culture plates and when 90 % of the cells were attaehed, the medium was replaced.

### HCECs stimulation assay

HCECs untreated were set as controls, anothers were added with *A. fumigatus* hyphea (5 × 10^6^/ml). Or HCECs were treated with 0.3 mg/ml laminarin, 5 μmol PLCγ2 inhibitor (U73122) or 10 μmol syk inhibitor (Piceatannol) for 30 min prior to *A. fumigatus* hyphae antigens stimulation in order to block Dectin-1, PLCγ2 and syk. After 15 min or 8 h’ incubation, HCECs were harvested to detect the protein and mRNA expression by western blot and RT-PCR.

### Calcium imaging

For analysis of cytoplasmic calcium, HCECs which seeded on the glass-bottom culture dishes (NEST) were labeled with Fluo-3 AM (5 μM;AAT Bioquest) for 60 min.To block PLCγ2, Fluo-3-loaded HCECs were preincubated with U73122 for 30 min at room temperature. After resting for 30 min, cells were stimulated with *A. fumigatus* hyphea(5 × 10^6^/ml) and cytoplasmic calcium was monitored on a Leica TCS SPE confocal microscope in real time for 6–8 min. The images were acquired using Leica LAS software before and after *A. fumigatus* hyphea were added for each condition. The fluoresecence intensity were measured using Image J software [21].

### Western blot

Cells were lysed in RIPA buffer for 1 h, and then were centrifuged. After estimation of protein content, addition of SDS sample buffer, and boiling, total protein was separated on 10% acrylamide SDS-PAGE and transferred onto a polyvinylidene difluoride membrane. The membranes were blocked with 5% BSA liquid, and then were incubated with a monoclonal antibody to human β-actin, and a monoclonal antibody to human Primary antibody at 4 °C overnight. After washed in PBST for three times, the membranes were incubated with corresponding peroxidase-conjugated secondary antibodies at 37 °C for 1 h. Then the blots.

were developed using chemiluminescence (ECL; Thermo Scientific).

### Real-time PCR

RNAiso plus reagent were used to extract total RNA from samples according to the manufacturer’s protocol, and the RNA was quantified by sprctrophotometry. The first strand cDNA was synthesized by RT from total RNA. The Real-Time PCR was performed in a Mx3005PTM system (Stratagene) with 20ul reaction volume containing 2ul of cDNA. cDNA was amplified by PCR using primers shown below. β-actin was used as the endogenous control. The thermocycler parameters were 95 °C for 30s, followed by 40 cycles of 95 °C for 5 s and 60 °C for 30s. A melting curve was used to confirm the specificity of the PCR products following each reaction. The ΔΔCT method was used for quantization of target gene.

products of stimulated and unstimulated group. Data are expressed as fold of increase in mRNA expressio. Each experiment was performed in triplicate. The double-stranded probes used are as follow: The following primers were used (5′-3′): AAGCCAGAGCTGTGCAGATGAGTA(forward) and TGTCCTGCAGCCACTGGTTC(reverse) for Human-IL-6; AGGGAATTCACCCCAAGAAC(forward) and CACCAGTGAGCTTCCTCCTC(reverse) for Human-CXCL1; TGCTTGTTCCTCAGCCTCTT(forward) and CAGAGGGCTGATTAGAGAGAGGT(reverse) for Human-TNF-α; TGGCACCCAGCACAATGAA(forward) and CTAAGTCATAGTCCGCCTAGAAGCA (reverse) for Human-β-actin as housekeeping gene.

### Enzyme-linked immunosorbent assay

According to the manufacturer’s protocol Enzyme-linked Immunosorbent Assay, Double-sandwich ELISA for human IL-6, CXCL1and TNF-α was performed, to detect the concentration of IL-6, CXCL1and TNF-α protein in conditioned media and culture cell lysates from different treatments. Absorbance was read at 450 nm with a reference wavelength of 570 nm by a VERSAmax microplate reader (Molecular Devices, Sunnyvale, CA) [21].

### Statistical analysis

All data were presented as mean ± SD from independent experiments. The data were analyzed using SPSS19.0 statistical package. One-way ANOVA test was used to make comparison among three or more groups, and LSD was used to identify between each two groups. *P* < 0.05 was considered statistically significant and data are shown as mean ± SEM.

## Results

### *A. fumigatus* induces cytokines production in a time- and dose-dependent manner

**Fig. 1:**
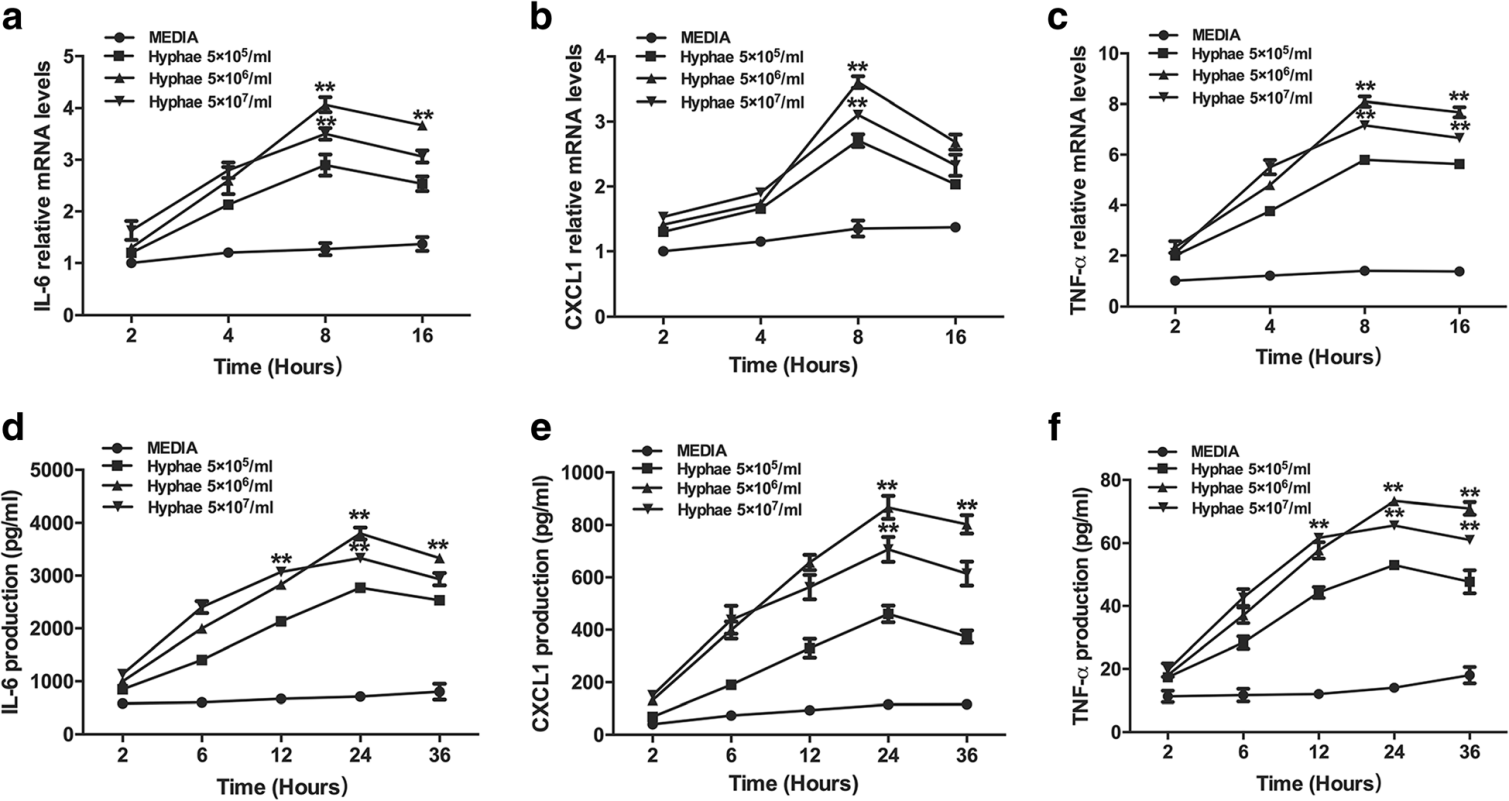
The effect of hyphae on mRNA and protein expression of IL-6, CXCL1 and TNF-α. **a-c** The RNA from HCECs treated with different concentrations of *A. fumigatus* hyphae (5 × 10^5^,5 × 10^6^ and5 × 10^7^ /ml) were harvested and tested by RT-PCR. **d-f** Cells were stimulated with *A. fumigatus* hyphae at 37 °C. The supernatants were collected and assayed by ELISA. The data represented the mean ± SEM of three separate experiments. **p < 0.001

To investigate *A. fumigatus* hyphae-induced IL-6, CXCL1 and TNF-α mRNA expression and protein secretion, hyphae-treated cells and supernatants were analyzed by RT-PCR and ELISA. The assys were performed over a period of 16 h and 36 h, with cells being exposed to 5 × 10^5^, 5 × 10^6^ and 5 × 10^7^/ml of hyphae, respectively. The level of IL-6, CXCL1 and TNF-α mRNA expression was elevated and peaked at 8 h (5 × 10^6^/ml), then returned to decrease after hyphae stimulation, as shown in Fig. 1a-c. The maximal protein production was recorded at 24 h (5 × 10^6^/ml) (Fig. 1d-f). The data demonstrated that IL-6, CXCL1 and TNF-α mRNA expression and protein secretion were induced by hyphae in a time- and dose-dependent manner in HCECs.

### PLCγ2 could be activated by *A. fumigatus* and induced by the engagement of Dectin-1 in HCECs

**Fig. 2:**
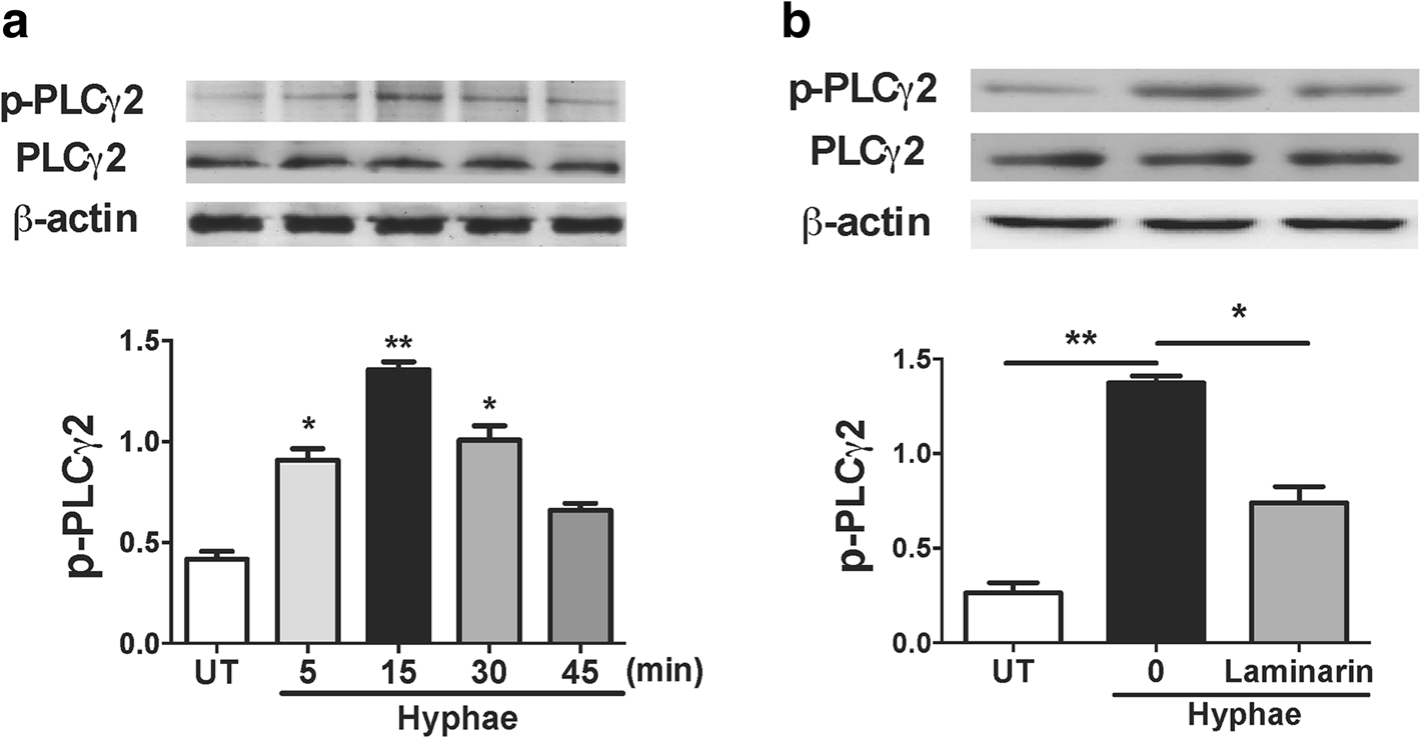
Engagement of Dectin-1 activates PLCγ2 in HCECs. **a** HCECs were treated with 5 × 10^6^/ml *A. fumigates hyphae* for various times as indicated their activation status determined by Western Blot. **b** HCECs were preincubated for 30 min with Dectin-1 inhibitor Laminarin, followed by the treatment of *hyphae* for 15 min. The activation of PLCγ was assayed by Western Blot. Data shown are representative of more than three independent sets of experiments. *p < 0.05, **p < 0.001

We stimulated HCECs with *A. fumigatus* hyphae, and the data showed that the stimulation led to the activation of PLCγ2 as indicated by their phosphorylation status. The phosphorylation of PLCγ2 was significantly increased after *A. fumigatus* hyphae infection with a time-dependent manner, and peaked at 15 min, whereas no significant difference was seen in total PLCγ2 protein (Fig. 2a). But we wondered whether the engagement of Dectin-1 could increase the activition of PLCγ2 in HCECs. To address this question, we preincubated HCECs with the Dectin-1 inhibitor laminarin before the stimulation of hyphae and data showed that this prior treatment could decrease the expression of p-PLCγ2 (Fig. 2b). Thus, it proved that the engagement of Dectin-1 played an important role in the activation of PLCγ2 with the stimulaton of hyphae in HCECs.

### PLCγ2 plays a role in the stimulation of Ca^2+^ flux induced by *A. fumigates*

**Fig. 3:**
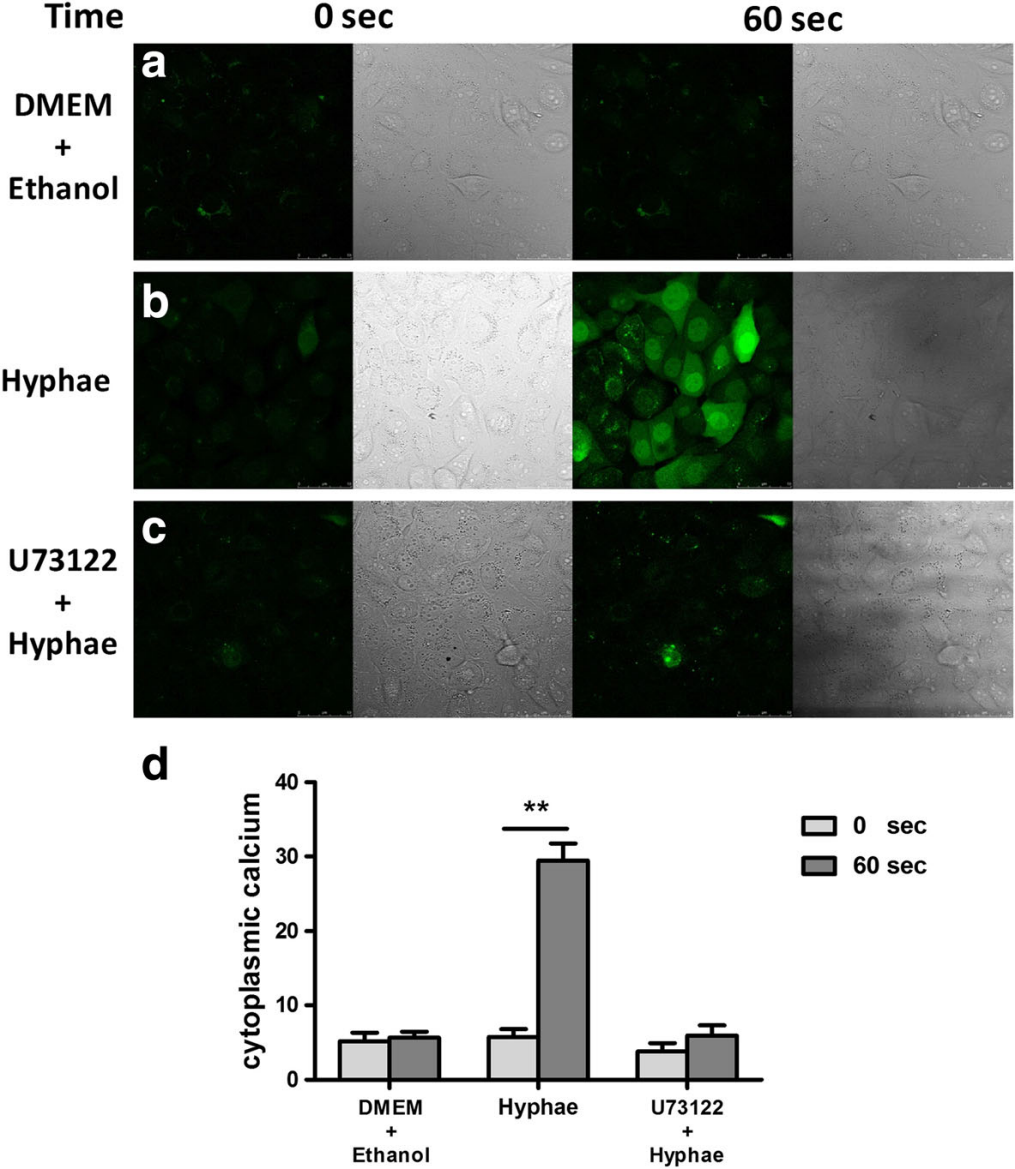
PLCγ2 plays an important role for the elicitation of Ca^2+^ flux in HCECs. **a-c** HCECs were loaded with Fluo-3 and treated with DMEM in the presence of Ethanol, 5 × 10^6^/ml *A. fumigates hyphae* or 5 × 10^6^/ml *A. fumigates hyphae* in the presence of 1μMol/L inhibitor of PLCγ2-U73122. Confocal images of HCECs showed cytoplasmic calcium expression (green stain). **d** The corresponding results of fluoresecence intensity were measured by Image J software. Data are representative of more than three independent sets of experiments. Magnifications 400X. **p < 0.001

To confirm whether PLCγ2 could induce Ca^2+^ flux in the infected HCECs, we preincubated the cells with U73122, the inhibitor of PLCγ2, prior to *A. fumigatus* hyphae treatment. As shown in Fig. 3, the Ca^2+^ flux elicited by treatment of hyphae in HCECs could be inhibited by U73122, which suggested that PLCγ2 played an important role in triggering Ca^2+^ signaling in HCECs.

### Syk is important for Dectin-1-induced PLCγ2 activation

**Fig. 4:**
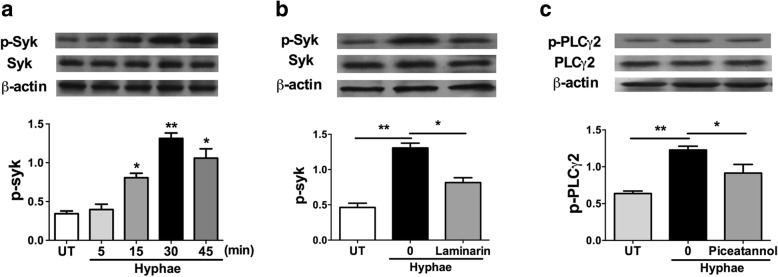
Syk Plays a crucial role for the activation of Dectin-1-induced PLCγ2 in HCECs. **a** The phosphorylation of Syk was activated after *A. fumigatus* hyphae stimulation in HCECs. The activation status was measured by Western Blot at 5, 15, 30 and 45 min. **b**, **c** HCECs were preincubated for 30 min with Dectin-1 inhibitor Laminarin or syk inhibitor Piceatannol, followed by treatment with hyphae for 30 or 15 min. The activation of syk or PLCγ was assayed by Western Blot. Results shown are mean ± SD of three independent experiments. *p < 0.05, **p < 0.001

To investigate the stimulatory effects on Syk with the *A. fumigatus* hyphae, the HCECs were incubated with the *A. fumigatus* hyphae (5 × 10^6^/mL) for 5, 15, 30 and 45 min, tested by western blotting. As shown in Fig. 4a, the findings indicated the phosphorylation of Syk was activated at 30 min by *A. fumigatus* hyphae stimulation in HCECs. We indicated that Dectin-1 was critical in the activation of Syk, because the inhibition of Dectin-1 abrogated hyphae-induced phosphorylation of Syk (Fig. 4b). To assess if the tyrosine kinase is important for Dectin-1-induced activation of PLCγ2, we pretreated HCECs with the Syk inhibitor Piceatannol(10 μM) for 30 min followed the stimulation of hyphae. Then we examined the phosphorylation status of PLCγ2. As shown in Fig. 4c, hyphae-stimulated phosphorylation of PLCγ2 was markedly reduced when Syk was inhibited by its specific inhibitor Piceatannol in HCECs. Consistent with the finding that Syk play important role in Dectin-1 signaling, our results further suggested that Syk acted upstream of PLCγ2 in the Dectin-1 signaling pathway as their activities are crucial for the activation of PLCγ2.

### PLCγ2 is essential for the up-regulation of cytokine production in HCECs

**Fig. 5:**
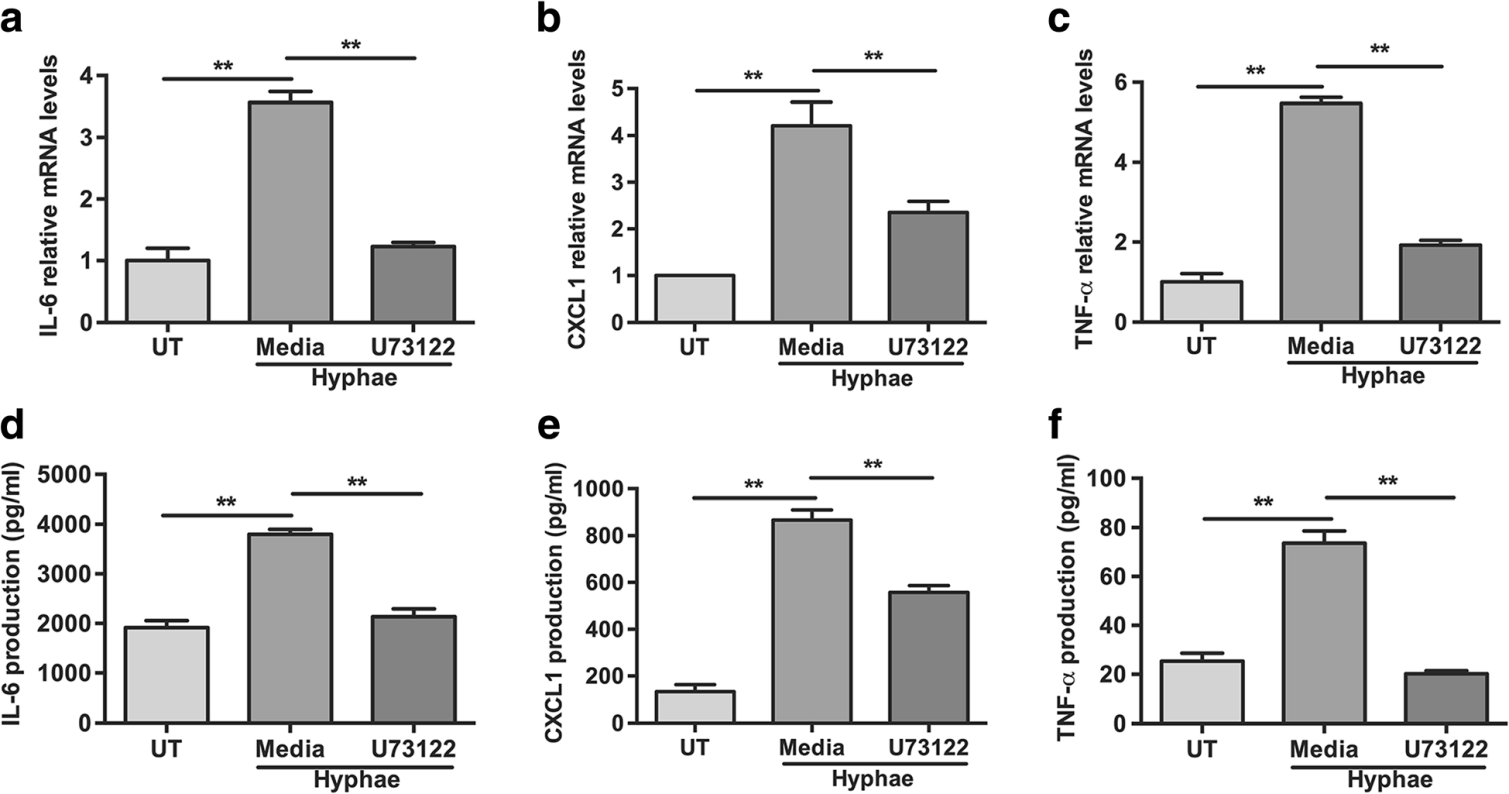
*A. fumigatus*-induced expression of inflammatory cytokines was regulated by PLCγ2 in HCECs. Cells were pre-treated with indicated concentrations of U73122 for 30 min prior to stimulation with 5 × 10^6^/ ml *A. fumigatus* hyphae. **a-c** mRNAs levels of IL-6, CXCL1 and TNF-α were reduced significantly after U73122 treatment. **d-f** Protein levels of these cytokines were also significantly reduced with the treatment of U73122. The data represented the mean ± SEM of three separate experiments. **P < 0.001

To determine whether PLCγ2 can regulate cytokine secretion upon *A. fumigatus* hyphae stimulation in HCECs, RT-PCR and ELISA were used to detect the expression of cytokine at 8 and 24 h. As seen in Fig. 5a-c, relative mRNA levels of IL-6, CXCL1 and TNF-α was significantly reduced with the pretreatment of U73122 compared with untreated controls. Protein analysis confirmed the mRNA data, with significant reduction between U73122 treated groups and controls (Fig. 5b-d). It suggested that PLCγ2 signaling critically regulated these cytokines in antifungal immunity.

## Discussion

FK is a blinding infection of the corneas, accounting for a significant portion in all keratitis. However, the pathogenesis of FK and the underlying molecular mechanisms are still unclear [24]. In the development of FK, innate immune response against *A. fumigatus* plays a crucial role in controlling microbial infection with the participation of inflammatory mediators [19]. In this study, we demonstrated that Dectin-1 mediated PLCγ2 signaling plays a critical role in *A. fumigatus* hyphae induced innate immune response in HCECs, and Syk acts as an upstream mediator in the Dectin-1 PLCγ2 signaling pathway. Above all, we showed that *A. fumigatus* hyphae significantly upregulated the expression of inflammatory factors IL-6, CXCL1 and TNF-α in HCECs. These fungi-induced cytokines resist fungal infection and promote the infiltration of inflammatory cells to remove pathogens.

In addition, our results suggested that PLCγ2 plays an important role in the stimulation of Ca^2+^ flux induced by *A. fumigates* in HCECs, which is consistent with that the enzymatic activity of PLCγ2 is required for the induction of Ca^2+^ flux in B cells [10]. Cytoplasmic Ca^2+^ flux is the hallmark of B cell receptor (BCR) signaling pathway, in which the engagement of PRRs is essential for the elicitation of Ca^2+^ flux in lymphocytes [9]. It is in the agreement with our previous study that the innate PRRs such as Dectin-1 could elicit intracellular Ca^2+^ flux [21]. It is also consistent with the importance of PLCγ2 for intracellular Ca^2+^ flux with the engagement of Dectin-1in DCs [25]. Recently, Lu et al. showed that the maintaince of intracellular Ca^2+^ homeostasis was involved in the potential mechanisms of hypoxia-induced inflammation and apoptosis in microglia BV2 cells [26]. Besides functioning in BCR signaling, PLCγ2 also plays an critical role in the innate immune system as a key component of the downstream signaling pathway for many receptors in response to fungal infection [11]. A previous study has shown that PLCγ2 functions downstream of Dectin-2 in response to the stimulation by the hyphal form of *Candida albicans* (*C. albicans*), an opportunistic pathogenic fungus [11]. In addition, they found that PLCγ2-deficient mice are defective in clearing *C. albicans* infection in vivo [11]. In this study, our findings showed that the lack of Dectin-1 impaired the phosphorylation of PLCγ2 in response to the infection with *A. fumigatus*, suggesting that Dectin-1 mediated the activation of PLCγ2 in the infected HCECs. Taken together, our data suggested that the intracellular Ca^2+^ mobilization, as a mechanism of cellular signaling in HCECs, is elicited by Dectin-1-mediated PLCγ2 signaling pathway.

Moreover, Dectin-1 is one of C-type lectin receptors (CLRs), functioning as PRRs to sense fungal infection. However, Dectin-1 induced PLCγ2 signaling pathway remains largely unknown. Here we explored how PLCγ2 was activated by determining which upstream kinase is required for its activation in the Dectin-1 signal transduction pathway. Recent studies demonstrated that Syk played critical roles in Dectin-1 signaling in macrophages [19, 20]. While in HCECs, we indicated that Syk was identified as a critical component downstream of Dectin-1 signaling, because the inhibition of Dectin-1 abrogated hyphae-induced activation of Syk. During BCR signaling, Syk is important for PLCγ2 activation and Ca^2+^ flux in B cells [27, 28]. Consistent with the results of BCR signaling, we found that the hyphae-stimulated phosphorylation of PLCγ2 was markedly reduced after Syk inhibition by its specific inhibitor Piceatannol in HCECs. Taken together, these findings demonstrated that Syk plays a critical role in Dectin-1-induced PLCγ2 signaling pathways, governing antifungal innate immune responses in HCECs.

Futhermore, it is reported that the lack of PLCγ2 impaired cytokine production in response to infection with *C. albicans* in PLCγ2-deficient macrophages [11]. Its deficiency resulted in the defective activation of NF-κB and MAPK and in a significantly reduced production of reactive oxygen species (ROS) following fungal challenge [11]. In our study, inflammatory mediators (IL-6, CXCL1 and TNF-α) stimulated by *A. fumigatus* hyphae were markedly blocked by PLCγ2 inhibitor in HCECs, suggesting that Dectin-1-mediated PLCγ2 signaling pathway is involved in the innate immune response of HCECs against *A. fumigatus* hyphae. PLCγ2 activation is essential to the expression of inflammatory mediators. These findings demonstrated that inflammatory cytokines and chemokines production could be upregulated through activation of Dectin-1-mediated PLCγ2 after *A. fumigatus* hyphae stimulation.

Last, but not least, it is increasingly acknowledged that C-type lectins act critically in the regulation of initiating and sustaining immune response against various pathogens. Therefore, the elucidation of the signaling of these CLRs would make a major impact on our understanding of host defense and microbial spread in the epithelium cells. For example, Dectin-1 is important for host recognition of β-glucan structure that exists in cell wall of *aspergillus*, *yeast*, *candida*, *penicillium* and other fungus [29–32]. Our current study demonstrated that PLCγ2 in Dectin-1 signal transduction could provide new targets for therapeutic intervention to enhance or suppress the host response.

## Conclusions

In conclusion, our findings demonstrate that PLCγ2 plays a critical role for Ca^2+^ Flux in HCECs stimulated by *A. fumigatus* hyphae. Syk acts upstream of PLCγ2 in the Dectin-1 signaling pathway. The expressions of proinflammatory mediators induced by *A. fumigatus* are regulated by the activation of Dectin-1-mediated PLCγ2 signaling pathway in HCECs. The further study of PLCγ2 pathway will provide new targets for the prevention and therapeutic intervention of fungal infection.

## Abbreviations

*A. fumigatus*: Aspergillus fumigatus
BCR: B cell receptor
*C. albicans*: *Candida albicans*
CLR: C-type lectin receptor
EGF: Epidermal Growth Factor
ELISA: Enzyme-linked immunosorbent assay
FBS: Fetal bovine serum
FK: Fungal keratitis
HCECs: Human corneal epithelial cells
MAPKs: Mitogen-activated protein kinases
NF-κB: Nuclear factor κB
PAMP: Pathogen associated molecular pattern
PBS: Phosphate buffere saline
PLCγ2: Phospholipase C-γ2
PRRs: Pattern-recognition receptors
RT-PCR: Reverse transcriptase polymerase chain reaction
Syk: Spleen-tyrosine kinase
